# Effect of Isoleucine and Added Valine on Performance, Nutrients Digestibility and Gut Microbiota Composition of Pigs Fed with Very Low Protein Diets

**DOI:** 10.3390/ijms232314886

**Published:** 2022-11-28

**Authors:** Parniyan Goodarzi, Caitlyn Marie Wileman, Mohammad Habibi, Katherine Walsh, Julia Sutton, Cedrick Ndhumba Shili, Jianmin Chai, Jiangchao Zhao, Adel Pezeshki

**Affiliations:** 1 Department of Animal and Food Sciences, Oklahoma State University, Stillwater, OK 74078, USA; 2 Department of Animal Science, Division of Agriculture, University of Arkansas, Fayetteville, AR 72701, USA

**Keywords:** very low protein diets, valine, isoleucine, nutrients digestibility, gut microbiota

## Abstract

Little is known whether a combination Ile and added Val improves the growth of pigs offered very low protein (VLP) diets through changes in nutrients digestibility and gut microbiota. The objective of this study was to investigate the effect of a mixture of Val above and Ile at NRC levels on growth, nutrient digestibility and gut microbiota in pigs fed with VLP diets. Forty, weaned piglets were assigned to: positive control: normal-protein-diet; negative control (NC): VLP diet supplemented with first four limiting amino acids; VA: NC with Val above NRC; IL: NC with Ile at NRC level; VAIL: NC with Val above and Ile at NRC levels. While both VAIL and VA groups completely recovered the inhibitory effects of VLP diets on feed intake, only VAIL partially recovered the negative effects of VLP diets on growth performance. VAIL and VA increased the thermal radiation and decreased the digestibility of nitrogen. NC increased the relative abundance of *Pasteurellaceae* and *Enterobacteriaceae* in the colon. VAIL had a higher abundance of colonic *Actinobacteria, Enterococcus*, and *Brevibacillus* and the colon content of VA was more enriched with *Mogibacterium*. Overall, VAIL partially improved the growth performance which is likely linked with alterations in gut microbiota composition.

## 1. Introduction

High protein diets have been criticized to be used for swine due to their negative impact on the environment, diet cost, post-weaning diarrhea and human and animal health [1]. Slightly low protein (SLP) diets with less than 4% reduced crude protein (CP) supplemented with first four limiting amino acids (FFL), i.e., lysine (Lys), methionine (Met), threonine (Thr) and tryptophan (Trp) reduce nitrogen (N) excretion, and post-weaning diarrhea and improve gut health [2,3] with no negative influence on growth performance of pigs [4,5,6,7]. Reduction of dietary CP more than 4% may produce even more beneficial results in total N excretion [8,9,10], but very low protein (VLP) diets reduce the growth performance of pigs while supplemented with FFL [11,12,13,14]. Further research is warranted to identify the next limiting amino acids (AA) in pigs fed with VLP diets. 

We have previously shown that supplementing VLP diets with all three branched-chain amino acids (BCAA) including leucine (Leu), isoleucine (Ile), and valine (Val) at or above NRC [15] levels along with adding FFL partially reversed the negative effect of these diets on growth of pigs [16,17]. Val and Ile have already been recommended as the fifth and sixth limiting AA for growth, respectively in growing-finishing pigs fed with VLP diets [18,19]. Valine deficiency has been shown to reduce the feed intake (FI) in 6-week-old pigs fed with VLP diet supplemented with FFL and histidine [20]. The reduction in growth following Val deficiency was more severe when these diets were supplemented with the excess amount of Leu [21]. Increasing dietary Leu levels has been shown to decrease daily FI and growth in weaned pigs fed with normal protein diets [22,23]. Therefore, dietary Val and Ile, but not Leu appear to have some promising effects on FI and growth of pigs under protein restriction.

We recently showed that supplementing a combination of Val and Ile at NRC [15] levels to VLP diets containing FFL improved growth performance of nursery pigs [24]. Others showed that dietary Val, and/or a combination of Val and Ile at NRC levels improved FI, growth performance and/or feed efficiency of pigs when added to both SLP [2,25,26,27] and VLP [18,28,29,30,31,32,33] diets containing FFL. However, adding Ile alone at NRC [15] levels to SLP and VLP diets supplemented with FFL either did not enhance the growth performance [2,25,28] or reduced FI and growth efficiency [18,29] in pigs. To our knowledge no study has examined the effect of a mixture of dietary Val above NRC [15] and Ile at NRC levels on growth of pigs offered VLP diets. 

Dietary BCAA enhance growth likely by improving intestinal development [24,34,35,36], FI [17,26,37,38,39], nutrients digestibility [40,41], AA utilization [42], insulin-like growth factor 1 (IGF-1) signaling [17], microbiota composition [16,43] and muscle protein synthesis [26,44,45,46]. The underlying mechanisms by which dietary Val, Ile and/or combination of both improve growth performance in pigs fed with VLP diets is poorly understood. Therefore, the objective of this study was to investigate the effect of Val above and Ile at NRC [15] levels on growth measurements, nutrients digestibility and gut microbiota in pigs fed with VLP diets.

## 2. Results

### 2.1. Growth Performance

**Figure 1:**
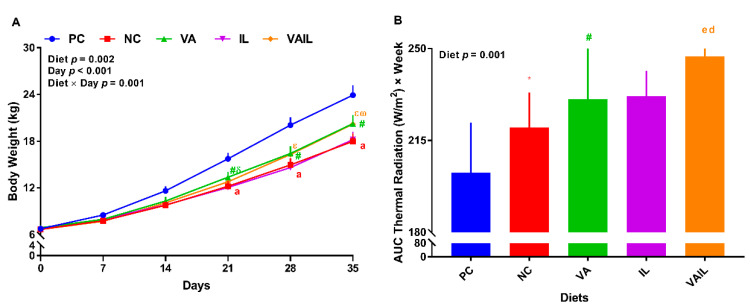
(**A**) Body weight, (**B**) area under the curve (AUC) for thermal radiation of nursery pigs fed with very low protein diets supplemented with isoleucine (Ile) at NRC and valine (Val) above NRC levels, or a combination of the two. PC: positive control, standard protein diet; NC: negative control, very low protein diet containing first four limiting amino acids (i.e., lysine, methionine, threonine and tryptophan) at NRC (2012) levels; VA: NC containing Val above NRC level; IL: NC containing Ile at NRC level; VAIL: NC containing Val above NRC and Ile at NRC levels. ^a^ *p* ≤ 0.05 NC vs. PC ^d^ *p* ≤ 0.05 VAIL vs. PC, ^e^ *p* ≤ 0.05 VAIL vs. NC, * *p* ≤ 0.1 NC vs. PC, ^#^ *p* ≤ 0.1 VA vs. PC, ^δ^ *p* ≤ 0.1 VA vs. NC, ^ε^ *p* ≤ 0.1 VAIL vs. PC, ^ω^ *p* ≤ 0.1 VAIL vs. NC. Values are means ± standard error of the mean. *n* = 8.

Overall, the effect of diet on final body weight (BW), average daily gain (ADG), average daily feed intake (ADFI), average daily protein intake (ADPI) and gain-to-feed (G:F) ratio was significant (*p* < 0.05, Table 1). Compared to PC, final BW for NC was reduced by 25%, while that tended (0.05 < *p* ≤ 0.1) to be reduced in VA and VAIL (Table 1). Relative to NC, VAIL, but not VA, tended to increase the final BW (Table 1). Pigs fed with NC, VA, and VAIL had 36, 24, and 22% lower ADG in comparison with PC, respectively. VAIL tended to increase the ADG compared to NC (Table 1). Body length, heart girth and wither height were not changed across diets (Table 1). Overall, the effects of diet, day, and interaction of diet by day on daily BW were significant (*p* < 0.01, Figure 1A). NC had a lower BW than PC on days 21, 28, and 35 (22, 22, and 25%, respectively (Figure 1A). Relative to PC, VA tended to have a lower BW on days 21, 28, and 35; however, VA tended to have a higher BW than NC on day 21. VAIL tended to reduce BW compared to PC on days 28 and 35 but this group tended to have a higher BW than NC on day 35 (Figure 1A). Further, the effect of diet on weekly body weight gain (BWG) was significant for weeks 2–5 and tended to be significant for week 1 (Table 2). Compared to PC, pigs fed with NC had a lower BWG during all 5 weeks (26, 38, 41, 37, and 38%, respectively). In comparison with PC, pigs fed with VA either tended or had a significantly lower BWG in all 5 weeks. Pigs fed with VA had a higher BWG than NC in week 3 (Table 2). VAIL tended to have a lower BWG than PC in weeks 2 and 5 and had a lower (38%) BWG in week 3. However, compared to NC, VAIL had a higher (40%) and tended to have a higher BWG during weeks 4 and 5, respectively.

Pigs in NC group had a lower ADFI than PC, but the ADFI was not different when VA and VAIL groups were compared with PC (Table 1). Compared to NC, the ADFI was 31% higher in VA and tended to be higher in VAIL (Table 1). Average daily water intake (ADWI) and water-to-feed ratio (W:F) were not changed among diets (Table 1). The effect of diet on FI was significant on day 4 with a lower FI for VAIL than PC and NC at hour 9 (0.05 < *p* ≤ 0.1, Figure S1). Overall, the effect of diet on mean feed intake (MFI) and cumulative feed intake (CFI) was significant in weeks 3–5 (Table 2). Relative to PC, NC tended to reduce MFI and CFI in week 3. VA tended to increase MFI and CFI compared to NC in week 3. NC had a lower MFI and CFI than PC in week 4. VA had a higher MFI and CFI than NC in week 4 and VAIL tended to have a higher MFI and CFI than NC in week 4 (Table 2).

ADPI was lower in NC, VA, and VAIL (67, 27, and 50%, respectively) compared to PC (Table 1). Furthermore, ADPI was 38% higher in VA and tended to be higher in VAIL compared to NC (Table 1). Overall, the effect of diet on cumulative protein intake (CPI) was significant over the course of 5 weeks (Table 2). In all weeks, pigs fed with NC had a lower CPI than PC. Except week 2, pigs fed with VA had a lower CPI than PC in weeks 1, 3, 4 and 5 (36, 33, 31, and 45%, respectively) (Table 2). However, compared to NC, pigs fed with VA increased CPI by 24 and 34% in weeks 3 and 4, respectively. Similarly, except week 2, pigs fed with VAIL had a lower CPI than PC in weeks 1, 3, 4 and 5 (40, 41, 30, and 27%, respectively) while, in comparison with NC, VAIL had 36 and 32% higher CPI in weeks 4 and 5, respectively.

Compared to PC, pigs fed with NC, VA, and VAIL had 26, 29, and 29% lower G:F ratio, respectively (Table 1). The effect of diet on weekly G:F ratio was significant on weeks 3 and 4 (Table 2). Pigs fed with NC had a lower G:F than PC in weeks 2, 4, 5. Relative to PC, pigs fed with VA decreased the G:F by 24, 23, and 31%, in weeks 2, 3, and 4, respectively and tended to reduce the G:F on week 5. VA tended to have a lower G:F than NC on week 4. In the last 3 weeks, G:F ratio in pigs fed with VAIL was lower in comparison with PC (23, 14 and 19%, respectively). The gain-to-protein ratio (G:P) was not different across diets (Table 1). The effect of diet on weekly G:P ratio was only significant on week 4 when pigs fed with VAIL had a higher (23%) G:P ratio than PC (Table 2).

### 2.2. Thermal Radiation

The effect of diet on area under the curve (AUC) of thermal radiation was significant (*p* < 0.01, Figure 1B). NC and VA tended to increase thermal radiation compared to PC. Pigs fed with VAIL had 22 and 12% higher AUC thermal radiation than PC and NC, respectively (Figure 1B).

### 2.3. Nutrients Digestibility

**Figure 2:**
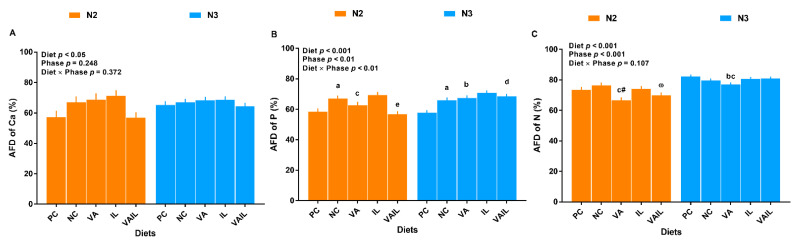
Apparent fecal digestibility (AFD) of (**A**) calcium (Ca), (**B**) phosphorus (P) and (**C**) nitrogen (N) in nursery pigs fed with very low protein diets supplemented with isoleucine (Ile) at NRC and valine (Val) above NRC levels, or a combination of the two. PC: positive control, standard protein diet; NC: negative control, very low protein diet containing first four limiting amino acids (i.e., lysine, methionine, threonine, and tryptophan) at NRC (2012) levels; VAIL: NC containing Val above NRC and Ile at NRC levels. ^a^ *p* ≤ 0.05 NC vs. PC, ^b^ *p* ≤ 0.05 VA vs. PC, ^c^ *p* ≤ 0.05 VA vs. NC, ^d^ *p* ≤ 0.05 VAIL vs. PC, ^e^ *p* ≤ 0.05 VAIL vs. NC, ^#^ *p* ≤ 0.1 VA vs. PC, ^ω^ *p* ≤ 0.1 VAIL vs. NC. The values are means ± standard error of the mean. *n* = 8.

There were no changes in apparent fecal digestibility (AFD) of calcium (Ca) across dietary treatments (Figure 2A). The overall effects of diet, phase, and diet by phase interaction on AFD of phosphorus (P) were significant (Figure 2B). Compared to PC, AFD of P was 15% higher in pigs fed with NC during N2 phase. The AFD of P in VA and VAIL was 6 and 16% lower than NC in N2 phase. In N3 phase, pigs fed with NC, VA, and VAIL had 14, 17 and 18% higher AFD of P than PC (Figure 2B). Overall, the effect of diet and phase on AFD of N was significant (Figure 2C). Pigs fed with VA reduced the AFD of N relative to NC in N2 and N3 phases. VA either tended to decrease or significantly decreased the AFD of N compared to PC in N2 and N3 phases, respectively. VAIL tended to have a lower AFD of N compared to NC in N2 phase (Figure 2C).

### 2.4. Dual-Energy X-ray Absorptiometry Analysis

Overall, the effect of diet on fat and lean percent was significant (*p* ≤ 0.01; Table 3). In comparison with PC, pigs fed with VA tended to have a higher fat percent in leg samples. Relative to NC, VA tended to have a lower lean percent in their leg. Bone mineral content (BMC) and bone mineral density (BMD) were not different across diets (Table 3). 

### 2.5. Colon Content Microbiota

**Figure 3:**
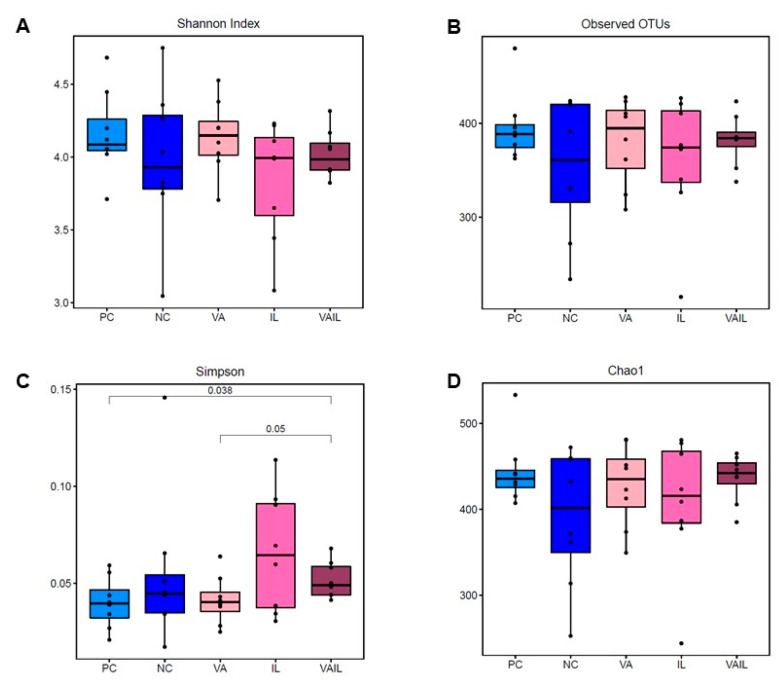
Alpha diversity indices for colon bacterial community in pigs fed with very low protein diets supplemented with isoleucine (Ile) at NRC and valine (Val) above NRC levels, or a combination of the two. (**A**) Shannon, (**B**) Observed operational taxonomic units (OTUs), (**C**) Simpson, and (**D**) Chao1. PC: positive control, standard protein diet; NC: negative control, very low protein diet containing first four limiting amino acids (i.e., lysine, methionine, threonine, and tryptophan) at NRC (2012) levels; VA: NC containing Val above NRC level; IL: NC containing Ile at NRC level; VAIL: NC containing Val above NRC and Ile at NRC levels. Each node represents an individual pig. The means are different with *p* ≤ 0.05. *n* = 8.

The rarefaction curve analysis showed that the species richness of all analyzed colon contents reached a stable plateau at 30,000 reads and 400 operational taxonomic units (OTUs) indicating that there was a sufficient sequencing depth to saturate the bacterial populations in samples (Figure S2A–E). The alpha diversity metrics of bacterial community in each sample are shown in Figure 3A–D. Simpson index, which shows the species diversity was significantly different in VAIL compared to PC and VA (*p* < 0.05; Figure 3C). No differences in Shannon, observed OTUs, and Chao1 were detected among groups (Figure 3A,B,D).

**Figure 4:**
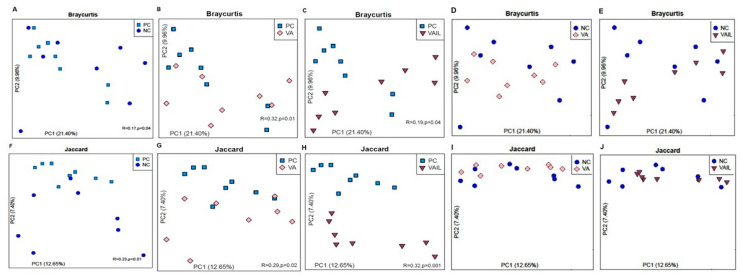
Beta diversity of colon bacterial community in pigs fed with very low protein diets supplemented with isoleucine (Ile) at NRC and valine (Val) above NRC levels, or a combination of the two. Principal coordinates analysis (PCoA) based on (**A**–**E**) Bray–Curtis and (**F**–**J**) Jaccard. (**A**,**F**) NC vs. PC, (**B**,**G**) VA vs. PC, (**C**,**H**) VAIL vs. PC, (**D**,**I**) VA vs. NC; (**E**,**J**) VAIL vs. NC. ANOSIM was used to test the difference of beta diversity between diet treatments. PC: positive control, standard protein diet; NC: negative control, very low protein diet containing first four limiting amino acids (i.e., lysine, methionine, threonine, and tryptophan) at NRC (2012) levels; VA: NC containing Val above NRC level; IL: NC containing Ile at NRC level; VAIL: NC containing Val above NRC and Ile at NRC levels. Each node represents an individual pig. *n* = 8.

The beta diversity of the bacterial community in colon contents among the groups is shown in Figure 4A–J. The Bray–Curtis and Jaccard distances showed significant separation and clustering for NC, VA, and VAIL compared to PC (*p* < 0.05; Figure 4A–C,F–H), which is suggestive of the differences in colon microbiota composition among pigs fed these diets. In comparison with NC, VA and VAIL groups showed no clear clustering (*p* > 0.05; Figure 4D,E,I,J).

**Figure 5:**
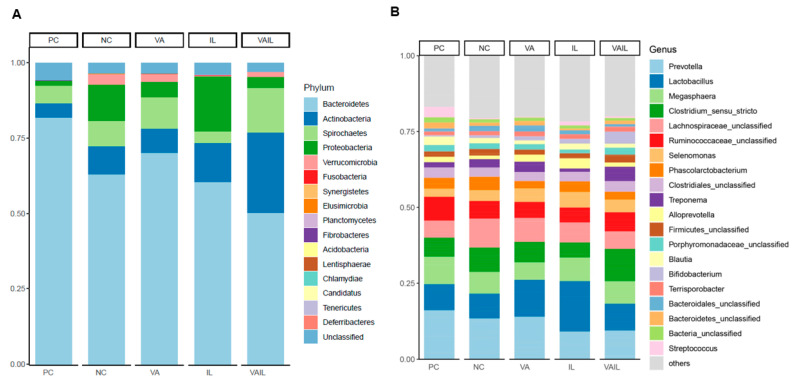
The relative abundance of colon bacterial composition at (**A**) phylum, and (**B**) genus levels in pigs fed with very low protein diets supplemented with isoleucine (Ile) at NRC and valine (Val) above NRC levels, or a combination of the two. PC: positive control, standard protein diet; NC: negative control, very low protein diet containing first four limiting amino acids (i.e., lysine, methionine, threonine, and tryptophan) at NRC (2012) levels; VA: NC containing Val above NRC level; IL: NC containing Ile at NRC level; VAIL: NC containing Val above NRC and Ile at NRC levels. *n* = 8.

Overall, at phylum level, Bacteroidetes, Actinobacteria and Spirochaetes and at genus level *Prevotella*, *Lactobacillus*, and *Megasphaera* were the most abundant bacteria in all diets (Figure 5A,B). The relative abundance of colon bacterial composition for individual pigs is shown in Figure S3.

**Figure 6:**
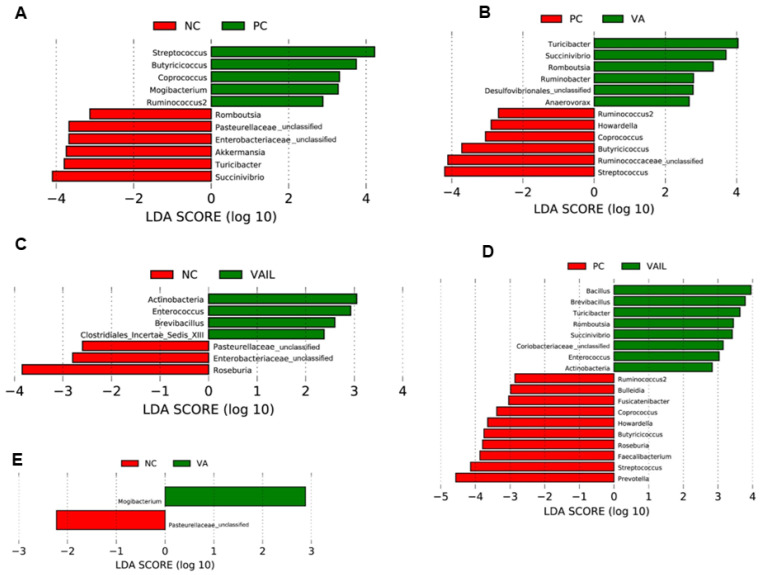
Histograms of colon microbiota composition in pigs fed with very low protein diets supplemented with isoleucine (Ile) at NRC and valine (Val) above NRC levels, or a combination of the two using linear discriminant analysis (LDA) with effect size (LEfSe). (**A**) NC vs. PC, (**B**) VA vs. PC, (**C**) VAIL vs. NC, (**D**) VAIL vs. PC, (**E**) VA vs. NC. PC: positive control, standard protein diet; NC: negative control, very low protein diet containing first four limiting amino acids (i.e., lysine, methionine, threonine, and tryptophan) at NRC (2012) levels; VA: NC containing Val above NRC level; IL: NC containing Ile at NRC level; VAIL: NC containing Val above NRC and Ile at NRC levels. *n* = 8.

Linear discriminant analysis (LDA) with effect size measurements (LefSe) for bacterial communities between dietary groups are shown in Figure 6. In comparison with PC, pigs fed with NC had higher proportions of *Succinivibrio*, *Turicibacter*, *Akkermansia*, *Enterobacteriaceae*, *Pasteurellaceae,* and *Romboutsia* (Figure 6A). Further pigs fed with VA had a higher abundance of *Turicibacter*, *Succinivibrio*, *Romboutsia*, *Ruminobacter*, *Desulfovibrionales* and *Anaerovorax* relative to PC (Figure 6B). VAIL had a higher abundance of *Bacillus*, *Brevibacillus*, *Turicibacter*, *Romboutsia*, *Succinivibrio*, *Coriobacteriaceae*, *Enterococcus* and *Actinobacteria* in comparison with PC (Figure 6D). Relative to NC, pigs fed with VAIL had a higher abundance of *Actinobacteria, Enterococcus, Brevibacillus* and *Clostridiales_Incertae_ Sedis_ XIII* (Figure 6C). In comparison with NC, pigs fed with VA had a higher abundance of *Mogibacterium* (Figure 6E).

## 3. Discussion

Very low protein diets reduce the total N excretion [8,9,10], but they have an adverse effect on growth performance of pigs even when supplemented with FFL [11,12,13,14]. We and others have shown that supplementing BCAA, Val and a combination of Val and Ile at NRC levels partially improve the growth performance of pigs fed with VLP diets [16,18,24,28,29,30,31,32,33]. Little is understood whether a combination of Val above NRC levels and standard amount of Ile will have additive effects on growth of pigs offered VLP diets. This study revealed several significant findings: (1) supplementation of a combination of Val above and Ile at NRC [15] levels (VAIL) partially restored the adverse effect of VLP diets on growth; (2) VAIL and VA fully recovered the negative effects of VLP diets on FI, increased the thermal radiation and reduced the AFD of N which might contribute to partial or lack of complete recovery of growth in these groups; (3) the colon content of VAIL group had higher abundance of *Actinobacteria, Enterococcus*, and *Brevibacillus* and that of VA was more enriched with *Mogibacterium* while pigs fed NC had a higher *Pasteurellaceae* and *Enterobacteriaceae*, which all may contribute to observed growth efficiency in above mentioned groups. Overall VAIL improved the growth performance of pigs fed with VLP diets likely through changing the composition of gut microbiota. 

In line with previous studies [11,12,13,14] VLP diets supplemented with FFL had an adverse effect on FI and growth performance of pigs. While VA and VAIL completely recovered the FI of pigs fed with VLP diets, these groups partially improved the growth, which is in parallel with other studies [18,28,29,30,31,32,33]. We and others have previously shown that supplementation of VLP or SLP diets with BCAA or Val increased the transcript of orexigenic neuropeptide Y and agouti-related protein and decreased the anorexigenic proopiomelanocortin, melanocortin-4-receptor, and cocaine- and amphetamine regulated transcript in hypothalamus, which may contribute to improved FI in BCAA supplemented groups [17,26,38,39]. We recently showed that Val and Ile combination added at NRC [15] levels into VLP diets improved the growth performance to the levels seen in pigs received standard protein diet [24]. The partial, but not complete recovery in growth performance in VA and VAIL groups in this study might be due to AA imbalances resulted from higher ratio of Val:Ile or ratio of Val to other AA. Excess dietary Val causing AA imbalances seems to interfere with the supply of Ile. The higher level of dietary Val in VAIL and VA groups may increase the α-ketoisovalerate, a keto acid produced by degradation of Val, levels that can allosterically inhibit the branched-chain α-ketoacid dehydrogenase which is involved in the first step of all three BCAA catabolism [47]. Therefore, excess dietary Val may result in reduction of plasma concentration of Ile as reported previously [48]. Supplementation of Val higher than NRC levels into VLP diet increased fat percentage while lean percentage decreased in excised legs of pigs. It has been reported that Val supplemented in drinking water with high fat diet increased body weight, serum triglyceride level and white adipose tissue weight suggestive of the possible role of Val in stimulating lipogenesis and fat deposition [49]. Whole body composition analysis in pigs following supplementation of Ile and Val to low protein diets may provide further insights on the role of these AA in nutrients partitioning.

The AA imbalances caused by higher ratio of Val to Ile or other AA in pigs fed with VA and VAIL groups may also deteriorate the growth performance through influencing their energy and nutrients retention. In the current study, VA and VAIL groups increased the thermal radiation. This data is consistent with our previous study [24] showing that supplementation of VLP diet with combination of Val and Ile at NRC levels stimulated the energy loss and thermal radiation. The AA imbalances as a result of higher Val to other AA ratio is likely detected by sensors with the subsequent activation or inhibition of downstream pathways resulting in increased energy expenditure [50]. Due to the fact that some AA share the same transport system, it remains to be determined whether reduction of Leu and Ile or other AA due to higher level of dietary Val contribute to increased energy loss in VA and VAIL groups. In the present study, the AFD of N was reduced in VA and VAIL groups. Other studies showed that adding Val alone or in combination with Ile had either no effect on [2] or increased [51] N retention in pigs fed with SLP diet. The discrepancy in results of above-mentioned studies and the current study might be explained by different CP contents and the ratio of Val to other AA. Since Val was added at a greater level than NRC values in this study, the reduced AFD of N in the VA and VAIL groups might be linked to AA imbalances. It has been previously reported that AA imbalances can cause growth retardation and reduce the limiting AA and N utilization efficiency [52]. The limitation of this study is that the digestibility of nutrients was assessed by AFD method only. In future studies the standard ileal digestibility assessments of nutrients, particularly individual AA will give greater insights on the role of Ile and Val on true digestibility of minerals and AA in pigs.

Previous studies have suggested several mechanisms for the positive effects of BCAA on growth performance of pigs such as improving the gut development [24,34,35,36], FI [17,26,37,38,39], nutrients and AA utilization [40,41,42], insulin-like growth factor 1 (IGF-1) signaling [17], and muscle protein synthesis [26,44,45,46]. Further, we and others showed that the stimulatory effects of BCAA on growth may be associated with alteration in gut microbiota composition [16,43]. It is unclear whether partial improvement in growth in VA and VAIL groups in the current study is associated with changes in gut microbiota. In our study, Bacteroidetes, Actinobacteria and Spirochaetes at phylum level, and *Prevotella*, *Lactobacillus*, and *Megasphaera* at genus level were the three most abundant bacteria in the colon contents of all dietary groups. Similarly, we and others previously reported that Bacteroidetes, Actinobacteria and Spirochaetes together with Firmicutes were among the main phyla in the feces, cecal digesta and colonic contents of pigs [13,16,53,54,55,56,57]. Further, in line with our data others showed that the abundance of *Prevotella* in the gut is increased after weaning, which is likely due to their increased ability in digestion of plant-based diets containing hemicelluloses and xylans [13,55,58,59,60]. Likewise, *Lactobacillus and Megasphaera* have both been shown to play a role in carbohydrate fermentation [61,62]. Other studies have reported bacterial communities such as *Streptococcus* and *Lactobacillus* as most abundant communities in the gut microbiome of pigs [63,64]. These differences in the main populations of gut microbiome might be related to differences in pigs breed, age, feed, and husbandry. In the current study, the Simpson index for bacterial populations in the colon was different in VAIL when compared to PC and VA which is suggestive of differences in species diversity in these groups.

In this study, the relative abundance of *Pasteurellaceae*, and *Enterobacteriaceae* was higher in pigs fed with VLP diets. Similarly, others showed an increase in abundance of *Enterobacteriaceae* in colon of weaned pigs fed with low protein diets [65]. *Pasteurellaceae*, and some members of *Enterobacteriaceae* are pathogenic bacteria [66,67]. In a previous study an increase in abundance of *Enterobacteriaceae* in diarrhoeic pigs was shown during post-weaning period [68]. The abundance of *Enterobacteriaceae* and intestinal chronic inflammatory diseases have been reported to be positively correlated [69]. Opportunistic pathogens such as *Salmonella Typhimurium* and *enterotoxigenic Escherichia coli* are members of the *Enterobacteriaceae* family that can cause intestinal inflammation and post-weaning diarrhea in piglets [70,71,72]. These results suggest that pigs fed with VLP diets reduce the growth performance likely due to gastrointestinal disturbances related with increased abundance of pathogenic bacteria that are potentially involved in gut inflammation. Further research is required to determine whether adverse effects of VLP diets on growth performance is associated with changes in certain inflammatory markers in post weaned pigs.

The colon content of pigs offered VAIL had higher abundance of *Actinobacteria*, *Enterococcus*, and *Brevibacillus* and that of VA was more enriched with *Mogibacterium*. *Actinobacteria* has been the focus of many studies due their importance for the maintenance of gut homeostasis as well as their potential for therapeutic use for gastrointestinal pathological conditions and systemic diseases [73]. The abundance of *Actinobacteria* has been reported to be decreased in patients with coeliac disease [74] or irritable bowel syndrome [75]. Therefore, *Actinobacteria* are thought to play an important role in health [76]. *Enterococcus faecium* has been used as a probiotic supplement in pigs’ diet with beneficial effects on reducing diarrhea and pathogenic *E. coli* in the intestine and improving growth performance and microbiota composition [77,78,79]. It appears that *Enterococci* improve growth performance either through producing organic acids that results in lowering pH and suppressing the pathogen strains in the gut [80] or inhibiting the pathogen’s adhesion to the intestinal mucosa [78]. *Brevibacillus brevis* is a unique candidate with broad-spectrum antimicrobial action [81] and has the potential to improve the growth performance and gut microbiota composition in pigs [82]. Previous studies have reported a positive link between the abundance of *Mogibacterium* and short-chain fatty acids concentration in feces of weaned pigs suggestive of a possible health benefit in these animals [83]. Overall, these data indicate that the improvement in growth performance of pigs fed with VAIL and VA might be due to probiotic and antibacterial properties and health benefits of their highly abundant colonic bacteria, *i.e., Actinobacteria*, *Enterococcus*, *Brevibacillus* and *Mogibacterium*.

## 4. Materials and Methods

### 4.1. Animals and Housing

All the experimental procedures used in this study were approved by Institutional Animal Care and Use Committee (IACUC-20-54) at Oklahoma State University. Forty weaned barrows (Duroc sire line and Large White × Landrace dam) weighing on average 6.14 kg at 3 weeks of age (Seaboard, Hennessey, OK, USA) were individually housed in a controlled temperature facility. The room temperature was set at 30 °C in the first week and then gradually decreased to 26 °C in the last week of the study. All pens were equipped with a single-hole stainless steel feeder and cup waterers (Aqua Chief, Newton Grove, NC, USA) attached to calibrated buckets with a single ½″ nipple (Lixit Nipple Waterer-L-70, Newton Grove, NC, USA). The lightening program was scheduled based on 12 h light and 12 h half-light (with lights on at 0800 and off at 2000). Throughout the study, all pigs had *ad libitum* access to feed and water. 

### 4.2. Diets and Experimental Design

Following 1 week of adaptation, pigs were weight matched (average body weight [BW] of 6.68 kg) and randomly assigned to 5 dietary treatments for 5 weeks as follows: (1) positive control (PC): normal protein diet; (2) negative control (NC): very low protein diet with Lys, Met, Thr and Trp (FFL) at NRC [15] levels; (3) VA: NC with Val above NRC level; (4) IL: NC with Ile at NRC level; (5) VAIL: NC with Val above and Ile at NRC level. National Swine Nutrition Guide (Version 2.1 Metric, ^©^2012 U.S. Pork Center of Excellence, Ames, IA, USA) was used for diet formulation. According to NRC recommendations, 3 nursery phase diets were prepared to ensure nutrients requirement of animals are met. Nursery phase 1 (N1), phase 2 (N2) and phase 3 (N3) were fed on days 1–7, 8–21 and 22–42, respectively. All diets were formulated to be isocaloric by using variable amounts of corn and soybean meal. Further, NC, Va, IL and VAIL diets were prepared isonitrogenous using L-Alanine. The amount of ingredients used was kept as consistent as possible among diets. The ingredients and chemical composition of diets are given in Table 4 and the analyzed chemical compositions of experimental diets are presented in Table 5.

### 4.3. Body Weight, Feed Intake, and Water Intake

Body weight were recorded weekly and individual FI and water intake (WI) were measured daily. Using BW, FI and WI data, ADG, ADFI, ADPI, ADWI, G:F, G:P and W:F were determined. Furthermore, BWG, MFI, CFI, CPI, G:F, and G:P ratios were calculated.

### 4.4. Thermal Images

Using a FLIR C2 compact thermal camera with a focal length of 1.54 mm and a thermal accuracy of ±2 °C (FLIR Systems, Boston, MA, USA), thermal images were captured approximately 1 m above each pig on a weekly basis with emissivity coefficient set at 0.95. Representative thermal images of experimental groups are shown in Figure S4.

### 4.5. Feed and Fecal Samples Collection

Roughly 1 kg feed samples from each feed bag were collected, pooled for each treatment, and stored at −20 °C until composition analysis. Fecal samples were collected in plastic bags for individual pigs over the course of study, combined separately during N2 and N3 phases for each treatment and stored at −20 °C for composition analysis.

### 4.6. Tissue Samples Collection

At week 6, after an overnight fast (8 h), pigs were allowed to consume their respective diets for one hour and at 120 min after meal, all pigs were euthanized using CO_2_ asphyxiation method. Immediately after euthanasia colon contents were collected, snap-frozen in liquid nitrogen, and stored at −80 °C for microbiota analyses. One of back legs was excised and stored at −20 °C for dual-energy X-ray absorptiometry (DEXA) analysis.

### 4.7. Diets and Fecal Samples Composition Analysis

Diets were analyzed for dry matter, CP, crude fiber, Ca, P, and chromium (Cr), by ServiTech laboratories (Dodge City, KS, USA) as we previously described [13,17,52] (Table 4). Experimental diets were also analyzed for complete AA profile by Agricultural Experiment Station Chemical Laboratories (University of Missouri-Columbia, Columbia, MO, USA) [84] (Table 5). N, Ca, P and Cr contents of fecal samples were determined by ServiTech laboratories (Dodge City, KS, USA).

### 4.8. Thermal Radiation Analysis

A rectangle shape was drawn in the entire back of piglets using FLIR camera software (FLIR Research Studio software) to determine the mean dorsal surface body temperature [48]. Following equation was then used to obtain thermal radiation (W/m^2^): σε (T_s_^4^–T_α_^4^) where σ is Stefan Boltzmann constant (5.67 × 10^−8^ W/m^2^K^4^), ε is thermodynamic emissivity (0.95), T_s_ is body surface temperature (kelvin) and T_α_ is ambient temperature (kelvin).

### 4.9. Apparent Fecal Digestibility

Marker method was used for calculating AFD of Ca, P, and N by comparing the difference between the quantities of Cr and nutrients in feed and feces for individual pigs using the following formula: AFD = 100 − [(100 × (Cr concentration in feed/Cr concentration in feces) × (nutrient concentration in feces/nutrient concentration in feed)] [53].

### 4.10. Dual-Energy X-ray Absorptiometry Analysis

Using rodent’s calibration feature, excised legs were scanned with DEXA (Hologic, Discovery QDR Series, Bedford, MA, USA) to obtain BMC, BMD, fat mass, lean mass, and total mass [53].

### 4.11. Colon Contents DNA Isolation, Amplicon Sequencing, Sequence Data Analysis, and Taxonomic Classification

The DNA from colon contents were extracted using the Dneasy PowerLyzer PowerSoil Kit (Qiagen, Inc., Germantown, MD, USA) following the instructions of the manufacturer. The concentration of isolated DNA was determined using NanoDrop One (Thermo Fisher Scientific, Madison, WI, USA) and diluted to 10 ng/μL. The DNA samples with OD 260/280 of 1.8–2 was used for PCR amplification and microbial amplicon sequencing.

For amplicon sequencing, the primers 515F (5′-GTGCCAGCMGCCGCGGTAA-3′) and 806R (5′-GGACTACHVGGGTWTCTAAT-3′) with barcodes were used for amplifying the 16S rRNA V4 region by PCR. The PCR products was mixed with 1× loading buffer containing GelRed and loaded on 1% electrophoresis agarose gel for quality control and then purified using normalization plates (SequalPrep Normalization Plate Kit, Invitrogen, Carlsbad, CA, USA). Purified PCR amplicons were pooled together to generate a sequencing library. The library quality and concentration were determined by using the Agilent Bioanalyzer 2100 system (Agilent, Santa Clara, CA, USA) and KAPA Illumina Library Quantification Kits (Roche, Indianapolis, IN, USA). A mock community (ZymoBIOMICS™ Microbial Community Standard, Zymo, Irvine, CA, USA) and negative control were included in the sequencing run for quality control to estimate errors introduced during PCR amplification and the MiSeq run. The library was then sequenced using the Illumina MiSeq platform (Illumina, Inc.), and 2 × 250 bp paired-end raw reads were generated.

For sequence data analysis, the Mothur software (v1.39.1, Ann Arbor, MI, USA) [85] was used to process the raw paired-end reads based on the MiSeq SOP. Paired-end reads were merged and filtered. Chimeras was removed by the UCHIME algorithm. Sequences were binned into OTUs at the 97% similarity level and be classified using a naïve Bayesian classifier against Ribosomal Database Project (RDP) [86]. High quality reads were classified against the RDP database. Alpha diversities including Shannon index, the number of Observed OTUs, Chao1 and Simpson were compared using Wilcoxon rank test. Beta diversity based on Bray–Curtis and Jaccard distances was tested using an analysis of similarity (ANOSIM). The outputs of diversity were visualized using the “ggplot2” package in R (version 3.6.2). To identify the most notable bacterial communities between dietary groups, LDA with LefSe was applied using a tool hosted in the Galaxy (server) instance of Huttenhower lab (https://huttenhower.sph.harvard.edu/galaxy/; accessed on 26 February 2022) and the scores were normalized by log10. The bacterial populations with LDA score (log10) > 2 were considered as significantly increased numbers.

### 4.12. Statistical Analysis

Outlier test was first conducted for overall growth, cumulative hourly FI, thermal radiation, and all other data acquired from laboratory analyses, including DEXA. GLM analysis (IBM SPSS Statistics Version 23, Armonk, NY, USA) and a paired Student’s *t*-test followed by a Benjamini-Hochberg correction with 0.1 false discovery rate was then used to determine the differences among means for five preplanned comparisons: NC vs. PC, VA vs. PC, VA vs. NC, VAIL vs. PC, and VAIL vs. NC. The mixed analysis was performed for the hourly, daily, and weekly collected data, including FI, WI, BW, BWG, MFI, CFI, CPI, G:F, and G:P, with the diet, time, and diet by time interaction as fixed effects and the animal as a random variable in the model. The modeling of covariance structure for repeated measurements for each variable was conducted using the lowest quantities of fit statistics for corrected Akaike Information Criterion and Bayesian Information Criterion. Differences among treatments were considered significant at *p* ≤ 0.05 and a trend at 0.05 < *p* ≤ 0.10.

## 5. Conclusions

In this study, for the first time, the effect of Val above and Ile at NRC levels on growth performance, nutrients digestibility, and gut microbiota in pigs fed VLP diets was addressed. We demonstrate that supplementing VLP diets with a mixture of Ile and added Val, i.e., VAIL, fully recovered the feed intake while partially restored the growth performance of pigs likely through increasing the abundance of *Actinobacteria, Enterococcus*, and *Brevibacillus* in colon. The partial, but not complete recovery in growth performance in VAIL group is likely associated with AA imbalances causing increased thermal radiation and reduced AFD of N in this group. While more attention has been paid towards the dietary protein quantity in swine production, it is important to emphasize the discussion on dietary protein quality and content of individual amino acids including Ile and Val to characterize the diets that not only improve the growth performance but also reduce the nutrients excretion from swine production.

## Figures and Tables

**Table 1:** Growth performance of nursery pigs fed with very low-protein diets supplemented with isoleucine at NRC and valine above NRC levels, or a combination of the two.

Measurements ^1^	Diets ^1^					SEM ^2^	*p*-Value
	PC	NC	VA	IL	VAIL		
Initial BW, kg	6.76	6.69	6.89	6.68	6.68	0.12	0.98
Final BW, kg	23.92	17.98 ^a^	20.26 ^#^	18.21	20.19 ^ε,ω^	0.56	0.01
ADG, kg/day	0.50	0.32 ^a^	0.38 ^b^	0.33	0.39 ^d,ω^	0.02	<0.01
ADFI, kg/day	0.78	0.62 ^a^	0.81 ^c^	0.68	0.78 ^ω^	0.02	0.04
ADPI, kg/day	0.15	0.08 ^a^	0.11 ^b,c^	0.09	0.10 ^d,ω^	0.01	<0.01
ADWI, L/day	1.95	1.38	1.73	1.55	1.77	0.08	0.24
G:F, kg/kg	0.66	0.49 ^a^	0.47 ^b^	0.48	0.50 ^d^	0.02	<0.01
G:P, kg/kg	3.47	3.66	3.62	3.73	3.74	0.09	0.91
W:F, L/kg	2.68	2.11	2.31	2.28	2.28	0.10	0.54
Final body length, m	0.65	0.61	0.65	0.61	0.63	0.01	0.08
Final heart girth, m	0.62	0.57	0.59	0.58	0.61	0.01	0.11
Final wither height, m	0.42	0.41	0.42	0.40	0.41	0.004	0.31

^1^ PC: positive control, standard protein diet; NC: negative control, very low protein diet containing first four limiting amino acids (i.e., lysine, methionine, threonine, and tryptophan) at NRC (2012) levels; VA: NC containing valine (Val) above of NRC level; IL: NC containing isoleucine (Ile) at NRC level; VAIL: NC containing Val above NRC and Ile at NRC levels. BW: body weight; ADG: average daily gain; ADFI: average daily feed intake; ADPI: average daily protein intake; ADWI: average daily water intake; G:F: gain:feed ratio; G:P: gain:protein ratio; W:F: water:feed ratio. ^a^ *p* ≤ 0.05 NC vs. PC, ^b^ *p* ≤ 0.05 VA vs. PC, ^c^ *p* ≤ 0.05 VA vs. NC, ^d^ *p* ≤ 0.05 VAIL vs. PC, ^#^ *p* ≤ 0.1 VA vs. PC, ^ω^ *p* ≤ 0.1 VAIL vs. NC, ^ε^ *p* ≤ 0.1 VAIL vs. PC. The values are means, *n* = 8. ^2^ SEM: standard error of the mean.

**Table 2:** Growth performance of nursery pigs fed with very low-protein diets supplemented with isoleucine at NRC and valine above NRC levels, or a combination of the two.

	Diets ^1^					SEM ^2^	*p*-Value
	PC	NC	VA	IL	VAIL		
**BWG ^1^, kg**							
Week 1	1.48	1.09 ^a^	1.07 ^b^	1.32	1.09	0.06	0.08
Week 2	3.23	2.00 ^a^	2.35 ^#^	1.82	2.19 ^ε^	0.12	<0.01
Week 3	4.11	2.44 ^a^	3.05 ^b,c^	2.24	2.54 ^d^	0.15	<0.01
Week 4	4.33	2.74 ^a^	3.05 ^b^	2.56	3.83 ^e^	0.16	<0.01
Week 5	4.85	3.03 ^a^	3.44 ^b^	3.27	3.89 ^ε,ω^	0.17	0.01
**MFI ^1^, kg**							
Week 1	0.44	0.40	0.42	0.35	0.42	0.02	0.54
Week 2	0.65	0.55	0.63	0.48	0.59	0.03	0.21
Week 3	0.83	0.63 *	0.81 ^δ^	0.63	0.75	0.03	0.05
Week 4	0.96	0.73 ^a^	0.96 ^c^	0.72	0.94 ^ω^	0.03	<0.01
Week 5	1.03	0.80	0.94	0.80	1.06	0.04	0.02
**CFI ^1^, kg**							
Week 1	3.09	2.78	2.96	2.48	2.96	0.12	0.56
Week 2	4.56	3.86	4.40	3.34	4.15	0.21	0.20
Week 3	5.78	4.41 *	5.65 ^δ^	4.41	5.28	0.19	0.05
Week 4	6.70	5.11 ^a^	6.74 ^c^	5.05	6.58 ^ω^	0.21	<0.01
Week 5	7.22	5.58	6.59	5.63	7.44	0.25	0.02
**CPI ^1^, kg**							
Week 1	0.62	0.38 ^a^	0.40 ^b^	0.39	0.37 ^d^	0.02	<0.01
Week 2	0.91	0.53 ^a^	0.65 ^#,δ^	0.51	0.66	0.04	<0.01
Week 3	1.08	0.58 ^a^	0.72 ^b,c^	0.55	0.64 ^d^	0.04	<0.01
Week 4	1.25	0.64 ^a^	0.86 ^b,c^	0.63	0.87 ^d,e^	0.05	<0.01
Week 5	1.35	0.74 ^a^	0.84 ^b^	0.70	0.98 ^d,e^	0.05	<0.01
**G:F ^1^, kg/kg**							
Week 1	0.51	0.42	0.36	0.72	0.41	0.07	0.46
Week 2	0.72	0.53 ^a^	0.55 ^b^	0.59	0.76	0.06	0.65
Week 3	0.71	0.56 *	0.55 ^b^	0.50	0.55 ^d^	0.02	0.01
Week 4	0.65	0.54 ^a^	0.45 ^b,δ^	0.51	0.56 ^d^	0.02	<0.01
Week 5	0.69	0.53 ^a^	0.53 ^#^	0.60	0.56 ^d^	0.02	0.11
**G:P ^1^, kg/kg**							
Week 1	2.54	3.06	2.69	3.06	3.29	0.12	0.31
Week 2	3.85	3.85	3.72	3.40	3.43	0.12	0.77
Week 3	3.82	3.79	4.27	4.05	4.20	0.11	0.56
Week 4	3.45	3.99	3.51	3.80	4.24 ^d^	0.09	0.04
Week 5	3.70	3.71	4.13	4.55	4.41	0.13	0.13

^1^ PC: positive control, standard protein diet; NC: negative control, very low protein diet containing first four limiting amino acids (i.e., lysine, methionine, threonine, and tryptophan) at NRC (2012) levels; VA: NC containing valine (Val) above of NRC level; IL: NC containing isoleucine (Ile) at NRC level; VAIL: NC containing Val above NRC and Ile at NRC levels. BWG: body weight gain; MFI: mean feed intake; CFI: cumulative feed intake; CPI: cumulative protein intake; G:F: gain:feed ratio; G:P: gain:protein ratio. ^a^ *p* ≤ 0.05 NC vs. PC, ^b^ *p* ≤ 0.05 VA vs. PC, ^c^ *p* ≤ 0.05 VA vs. NC, ^d^ *p* ≤ 0.05 VAIL vs. PC, ^e^ *p* ≤ 0.05 VAIL vs. NC, * *p* ≤ 0.1 NC vs. PC, ^#^ *p* ≤ 0.1 VA vs. PC, ^δ^ *p* ≤ 0.1 VA vs. NC, ^ε^ *p* ≤ 0.1 VAIL vs. PC, ^ω^ *p* ≤ 0.1 VAIL vs. NC. The *p*-values for the overall model effect for diet, week (wk) and diet × wk for BWG were <0.01, <0.01 and <0.01, for MFI were <0.01, <0.01 and 0.169, for CFI were <0.01, <0.01 and 0.607, for CPI were <0.01, <0.01 and 0.025, for G:F were 0.180, 0.084 and 0.515 and for G:P were 0.092, <0.01 and 0.427, respectively. The values are means, *n* = 8. ^2^ SEM: standard error of the mean.

**Table 3:** Dual-energy X-ray absorptiometry (DEXA) scan of nursery pigs’ leg fed with very low-protein diets supplemented with isoleucine at NRC and valine above NRC levels, or a combination of the two.

Measurements	Diets ^1^					SEM ^2^	*p*-Value
	PC	NC	VA	IL	VAIL		
Fat, %	12.70	13.80	16.35 ^#^	19.69	14.77	0.62	<0.01
Lean, %	84.97	83.71	81.01 ^δ^	77.99	82.94	0.64	<0.01
BMC ^1^, g	47.09	39.65	45.78	39.10	41.23	1.58	0.38
BMD ^1^, g/cm^2^	0.31	0.29	0.31	0.29	0.30	0.01	0.61

^1^ PC: positive control, standard protein diet; NC: negative control, very low protein diet containing first four limiting amino acids (i.e., lysine, methionine, threonine, and tryptophan) at NRC (2012) levels; VA: NC containing valine (Val) above of NRC level; IL: NC containing isoleucine (Ile) at NRC level; VAIL: NC containing Val above NRC and Ile at NRC levels. BMC: bone mineral content, BMD: bone mineral density. ^#^ *p* ≤ 0.1 VA vs. PC, ^δ^ *p* ≤ 0.1 VA vs. NC. The values are means, *n* = 8. ^2^ SEM: standard error of the mean.

**Table 4:** Experimental diets’ ingredients and calculated chemical composition (as-fed basis).

	Diets ^1^										
	N1	N2					N3				
Ingredients ^2^, %		PC	NC	VA	IL	VAIL	PC	NC	VA	IL	VAIL
Corn, yellow dent	36.98	46.35	67.62	67.49	67.50	67.35	66.20	84.04	83.93	83.94	83.82
Soybean meal, 47.5% CP	17.00	26.40	6.60	6.60	6.60	6.60	21.28	2.83	2.83	2.83	2.83
Fish meal, menhaden	6.00	3.20	3.20	3.20	3.20	3.20	3.20	3.20	3.20	3.20	3.20
Whey, dried	25.00	4.50	4.50	4.50	4.50	4.50	─	─	─	─	─
Corn starch	─	13.88	10.62	10.62	10.62	10.62	4.00	2.91	2.91	2.91	2.91
Lactose	7.00	─	─	─	─	─	─	─	─	─	─
Plasma spray-dried	5.80	2.10	2.10	2.10	2.10	2.10	2.10	2.10	2.10	2.10	2.10
Corn oil	0.37	─	─	─	─	─	─	─	─	─	─
Dicalcium phosphate 18.5%	0.85	1.35	1.73	1.73	1.73	1.73	1.15	1.55	1.55	1.55	1.55
Limestone	0.39	0.50	0.41	0.41	0.41	0.41	0.47	0.34	0.34	0.34	0.34
Salt	0.13	0.59	0.59	0.59	0.59	0.59	0.48	0.48	0.48	0.48	0.48
Chromium oxide	─	0.50	0.50	0.50	0.50	0.50	0.50	0.50	0.50	0.50	0.50
Vitamin premix	0.04	0.03	0.03	0.03	0.03	0.03	0.03	0.03	0.03	0.03	0.03
Trace mineral premix	─	0.01	0.01	0.01	0.01	0.01	0.01	0.01	0.01	0.01	0.01
Zinc oxide, 72% Zn	0.01	0.01	0.01	0.01	0.01	0.01	0.01	0.01	0.01	0.01	0.01
L-Lysine, HCl	0.26	0.35	0.85	0.85	0.85	0.85	0.36	0.83	0.83	0.83	0.83
DL-methionine	0.13	0.11	0.20	0.20	0.20	0.20	0.09	0.17	0.17	0.17	0.17
L-threonine	0.04	0.10	0.37	0.37	0.37	0.37	0.10	0.36	0.36	0.36	0.36
L-tryptophan	─	0.01	0.11	0.11	0.11	0.11	0.01	0.10	0.10	0.10	0.10
L-isoleucine	─	─	─	─	0.33	0.33	─	─	─	0.31	0.31
L-valine	─	─	─	0.48	─	0.48	─	─	0.44	─	0.44
L-alanine	─	─	0.54	0.19	0.33	─	─	0.53	0.20	0.32	─
**Calculated Chemical Composition** ^3^											
Dry matter, %	90.38	90.36	90.14	90.05	90.16	90.18	88.93	88.97	88.98	88.98	88.99
ME, Mcal/kg	3.40	3.40	3.40	3.40	3.40	3.40	3.33	3.34	3.34	3.34	3.34
Crude protein, %	21.97	20.30	14.00	14.00	14.00	14.00	18.93	12.93	12.93	12.93	12.93
Crude fiber, %	1.61	2.23	1.86	1.85	1.85	1.85	2.47	2.07	2.07	2.07	2.06
Crude fat, %	3.23	2.72	3.08	3.08	3.08	3.08	3.28	3.54	3.54	3.54	3.53
Calcium, %	0.85	0.80	0.80	0.80	0.80	0.80	0.70	0.70	0.70	0.70	0.70
Total phosphorus, %	0.70	0.65	0.65	0.65	0.65	0.65	0.60	0.60	0.60	0.60	0.60
Available phosphorus, %	0.62	0.46	0.51	0.51	0.51	0.51	0.39	0.44	0.44	0.44	0.44
SID Lysine, %	1.50	1.35	1.35	1.35	1.35	1.35	1.23	1.23	1.23	1.23	1.23
SID Threonine, %	0.88	0.79	0.79	0.79	0.79	0.79	0.73	0.73	0.73	0.73	0.73
SID Methionine, %	0.43	0.39	0.39	0.39	0.39	0.39	0.36	0.36	0.36	0.36	0.36
SID Tryptophan, %	0.25	0.22	0.22	0.22	0.22	0.22	0.20	0.20	0.20	0.20	0.20
SID Isoleucine, %	0.78	0.74	0.41	0.41	0.74	0.74	0.67	0.36	0.36	0.67	0.67
SID Valine, %	0.96	0.86	0.53	1.01	0.53	1.01	0.79	0.48	0.92	0.48	0.92
SID Leucine, %	1.65	1.50	1.05	1.05	1.05	1.05	1.46	1.03	1.03	1.03	1.02
SID Histidine, %	0.51	0.47	0.30	0.30	0.30	0.30	0.45	0.28	0.28	0.28	0.28
SID Arginine, %	1.13	1.19	0.62	0.62	0.62	0.62	1.09	0.55	0.55	0.55	0.55
SID Phenylalanine, %	0.90	0.86	0.51	0.51	0.51	0.51	0.80	0.47	0.47	0.47	0.47
SID Valine: SID Lysine	0.64	0.64	0.39	0.75	0.39	0.75	0.64	0.39	0.75	0.39	0.75
SID Isoleucine: SID Lysine	0.52	0.55	0.30	0.30	0.55	0.55	0.54	0.29	0.29	0.54	0.54

^1^ National Swine Nutrition Guide (Version 2.1 Metric, ©2012 U.S. Pork Center of Excellence, Ames, IA, USA) was used for diets formulations. PC: positive control, standard protein diet; NC: negative control, very low protein diet containing first four limiting amino acids (i.e., lysine, methionine, threonine, and tryptophan) at NRC (2012) levels; VA: NC containing valine (Val) above of NRC level; IL: NC containing isoleucine (Ile) at NRC level; VAIL: NC containing Val above NRC and Ile at NRC levels. N1: nursery phase 1 diet offered from days 1 to 7 of the experiment, N2: nursery phase 2 diets fed from days 8 to 21 of the experiment and N3: nursery phase 3 diets offered from days 22 to 42 of the experiment. ^2^ Corn, soybean meal, fish meal, whey, corn starch, lactose, plasma spray-dried, corn oil, dicalcium phosphate, limestone, salt, zinc oxide, DL-methionine (99%) and L-lysine HCl (79–99%) were obtained by Nutra Blend, LLC (Neosho, MO, USA). L-threonine (98.5%) and L-tryptophan (98%) were purchased from Ajinomoto (Overland Park, KS, USA). L-isoleucine (98.5%), L-alanine and L-valine (96.5%) were provided from Ajinomoto Health & Nutrition North America, Inc. (Raleigh, NC, USA). Chromium oxide was ordered from Fisher Scientific (Bartlesville, OK, USA). Vitamin premix was obtained from Nutra Blend, LLC (Neosho, MO, USA): vitamin A, 1,653,750 IU/kg; vitamin D3, 661,500 IU/kg; vitamin E, 17,640 IU/kg; vitamin K (menadione), 1323 mg/kg; vitamin B12, 13.23 mg/kg; niacin, 19,845 mg/kg; D-pantothenic acid, 11,025 mg/kg; riboflavin, 3307.5 mg/kg; phytase, 300,056.4 FYT/kg. Trace mineral premix was obtained from Nutra Blend, LLC (Neosho, MO): copper, 11,000 ppm; iodine, 198 ppm; iron, 73,000 ppm; manganese, 22,000 ppm; selenium, 198 ppm; zinc, 73,000 ppm. ^3^ ME: Metabolize energy; SID: Standard Ileal Digestibility.

**Table 5:** Analyzed chemical composition of experimental diets (as-fed basis).

	Diets ^1^										
	N1	N2					N3				
Chemical Composition		PC	NC	VA	IL	VAIL	PC	NC	VA	IL	VAIL
Dry matter, %	90.60	88.10	87.60	86.80	87.70	87.00	87.30	86.80	86.60	86.90	86.50
Crude protein, %	23.00	20.00	13.70	13.50	14.40	13.50	18.70	13.20	12.80	12.40	13.20
Crude fiber, %	1.40	2.00	1.70	1.80	1.90	1.40	2.90	2.00	1.90	1.90	2.00
Calcium, %	1.06	0.92	0.66	0.83	0.88	0.78	0.78	0.64	0.66	0.71	0.67
Phosphorus, %	0.94	0.76	0.67	0.74	0.79	0.70	0.61	0.61	0.67	0.69	0.69
Taurine ^2^, %	0.20	0.19	0.28	0.19	0.20	0.20	0.26	0.28	0.21	0.21	0.20
Hydroxyproline, %	0.11	0.09	0.05	0.06	0.10	0.08	0.06	0.06	0.09	0.06	0.06
Aspartic acid, %	2.10	2.09	1.02	1.06	1.30	0.93	1.81	0.88	0.89	0.94	0.97
Threonine, %	1.06	0.93	0.85	0.75	0.92	0.80	0.86	0.75	0.72	0.94	0.98
Serine, %	0.95	0.91	0.51	0.51	0.59	0.47	0.81	0.47	0.47	0.49	0.50
Glutamic acid, %	3.45	3.57	1.94	2.03	2.41	1.85	3.31	1.80	1.79	1.86	1.89
Proline, %	1.17	1.11	0.73	0.76	0.81	0.72	1.05	0.72	0.76	0.76	0.77
Lanthionine ^2^, %	0.02	0.01	0.02	0.00	0.00	0.00	0.02	0.02	0.00	0.00	0.00
Glycine, %	0.95	0.88	0.53	0.52	0.63	0.49	0.83	0.51	0.52	0.53	0.51
Alanine, %	1.14	1.02	1.29	0.97	1.08	0.69	1.00	1.19	0.84	1.21	0.72
Cysteine, %	0.44	0.39	0.23	0.24	0.29	0.21	0.33	0.23	0.20	0.24	0.26
Valine, %	1.18	1.04	0.61	1.03	0.74	1.04	0.94	0.55	0.98	0.60	0.97
Methionine, %	0.47	0.44	0.36	0.35	0.44	0.39	0.39	0.37	0.29	0.34	0.38
Isoleucine, %	0.93	0.91	0.49	0.50	0.89	0.75	0.81	0.43	0.41	0.74	0.71
Leucine, %	1.89	1.73	1.12	1.17	1.29	1.12	1.63	1.09	1.10	1.12	1.14
Tyrosine, %	0.73	0.68	0.39	0.39	0.47	0.39	0.63	0.39	0.39	0.38	0.39
Phenylalanine, %	1.01	1.02	0.56	0.60	0.69	0.54	0.92	0.53	0.54	0.53	0.56
Hydroxylysine, %	0.06	0.04	0.00	0.03	0.04	0.03	0.00	0.00	0.04	0.02	0.04
Ornithine ^2^, %	0.01	0.02	0.01	0.01	0.01	0.01	0.02	0.01	0.01	0.01	0.01
Lysine, %	1.69	1.69	1.39	1.32	1.56	1.31	1.40	1.29	1.37	1.40	1.29
Histidine, %	0.54	0.54	0.32	0.32	0.37	0.28	0.51	0.30	0.30	0.31	0.30
Arginine, %	1.19	1.29	0.65	0.65	0.81	0.56	1.16	0.58	0.59	0.61	0.60
Tryptophan, %	0.33	0.28	0.23	0.25	0.26	0.28	0.22	0.20	0.22	0.24	0.22
Valine: Lysine	0.70	0.62	0.44	0.78	0.47	0.79	0.67	0.43	0.72	0.43	0.75
Isoleucine: Lysine	0.55	0.54	0.35	0.38	0.57	0.57	0.58	0.33	0.30	0.53	0.55

^1^ PC: positive control, standard protein diet; NC: negative control, very low protein diet containing first four limiting amino acids (i.e., lysine, methionine, threonine, and tryptophan) at NRC (2012) levels; VA: NC containing valine (Val) above of NRC level; IL: NC containing isoleucine (Ile) at NRC level; VAIL: NC containing Val above NRC and Ile at NRC levels. N1: nursery phase 1 diet offered from days 1 to 7 of the experiment, N2: nursery phase 2 diets fed from days 8 to 21 of the experiment and N3: nursery phase 3 diets offered from days 22 to 42 of the experiment. ^2^ Non-proteinogenic amino acids.

## Data Availability

Datasets supporting the results of this article are included within the article and its supplementary files.

## Supplementary Materials

The following supporting information can be downloaded at: https://www.mdpi.com/article/10.3390/ijms232314886/s1.
