# Supplementary material for: Effect of Isoleucine and Added Valine on Performance, Nutrients Digestibility and Gut Microbiota Composition of Pigs Fed with Very Low Protein Diets
Source: Int J Mol Sci. 2022 Nov 28;23(23):14886. doi: 10.3390/ijms232314886 (PMC9740036; doi:10.3390/ijms232314886)
Supplement: Supplementary file 1 [file ijms-23-14886-s001.zip › ijms-2016930-supplementary.pdf]

Supplementary Material

Supplementary Figures

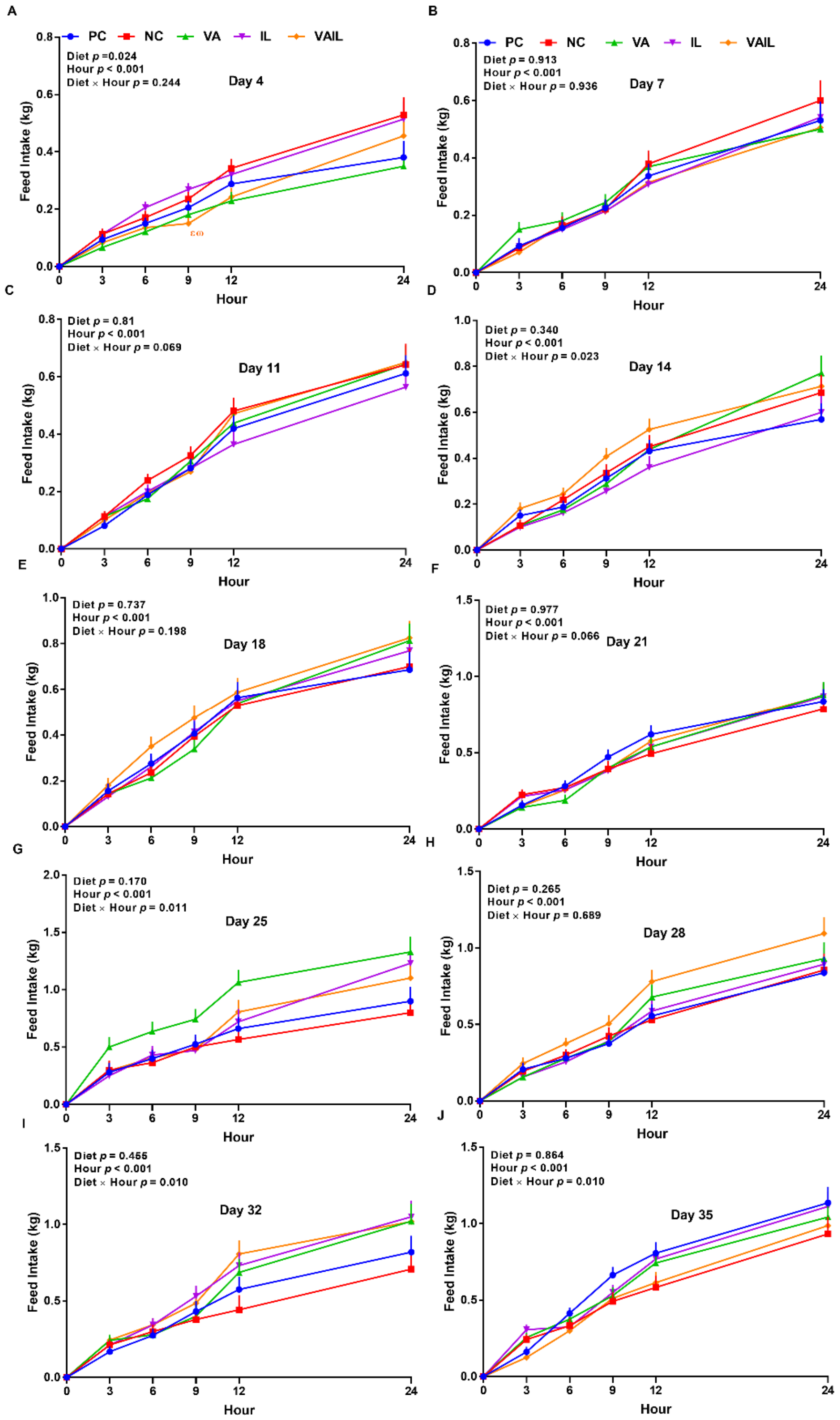

Figure S1. Feed intake at day (**A**) 4, (**B**) 7, (**C**) 11 (**D**) 14, (**E**) 18, (**F**) 21, (**G**) 25, (**H**) 28, (**I**) 32, and (**J**) 35 in nursery pigs fed with very low protein diets supplemented with isoleucine (Ile) at NRC and valine (Val) above NRC levels, or a combination of the two. PC: positive control, standard protein diet; NC: negative control, very low protein diet containing first four limiting amino acids (*i.e.*, lysine, methionine, threonine, and tryptophan) at NRC (2012) levels; VA: NC containing Val above NRC level; IL: NC containing Ile at NRC level; VAIL: NC containing Val above NRC and Ile at NRC levels. $^{\omega}\ p \leq 0.1$ VAIL vs. NC, $^{\varepsilon}\ p \leq 0.1$ VAIL vs. PC. The values are means ± standard error of the mean. n=8.

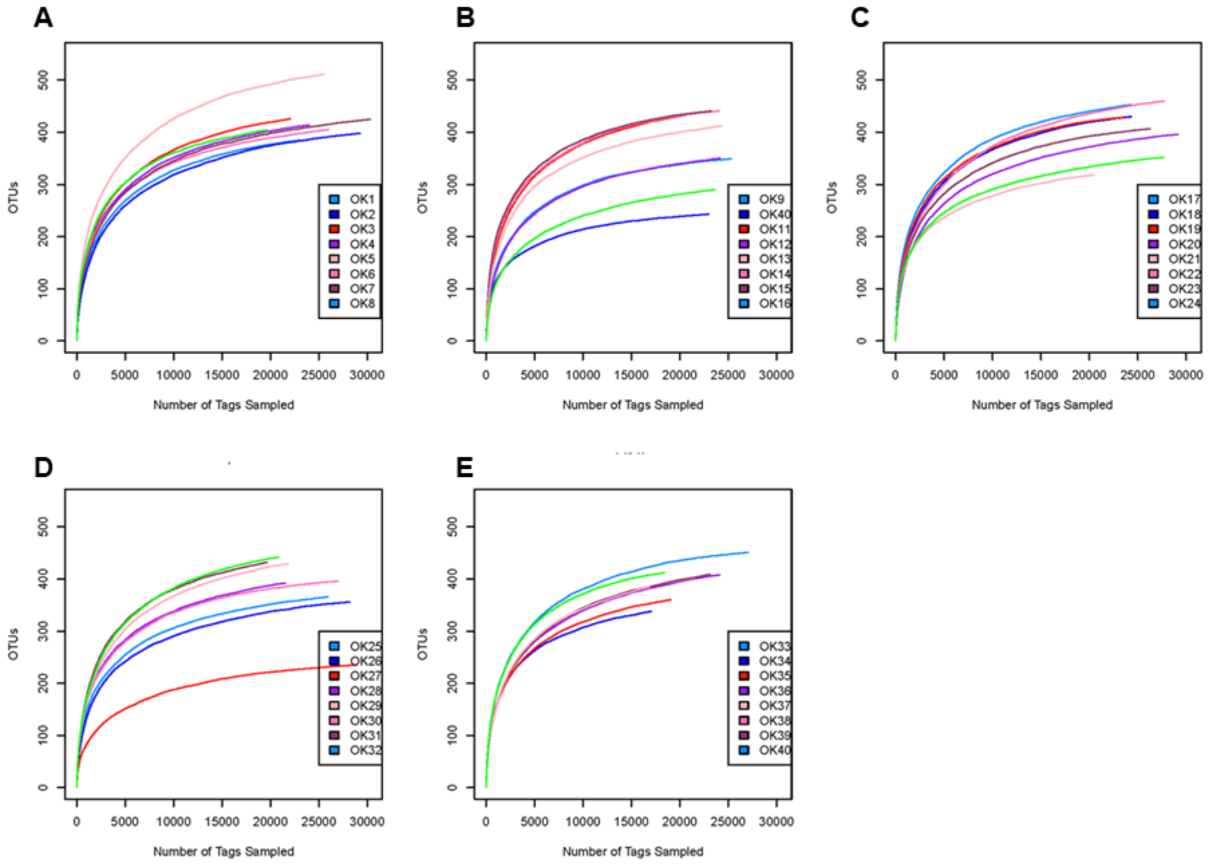

Figure S2. Rarefaction curve analysis for colon samples collected from nursery pigs fed with very low protein diets supplemented with isoleucine (Ile) at NRC and valine (Val) above NRC levels, or a combination of the two. The rarefaction curves illustrate the number of operational taxonomic units (OTUs) discovered as a function of the number of reads sampled when data were evaluated for individual animals. (**A**) PC, (**B**) NC, (**C**) VA, (**D**) IL, and (**E**) VAIL PC: positive control, standard protein diet; NC: negative control, very low protein diet containing first four limiting amino acids (*i.e.*, lysine, methionine, threonine, and tryptophan) at NRC (2012) levels; VA: NC containing Val above NRC level; IL: NC containing Ile at NRC level; VAIL: NC containing Val above NRC and Ile at NRC levels. *n* = 8.

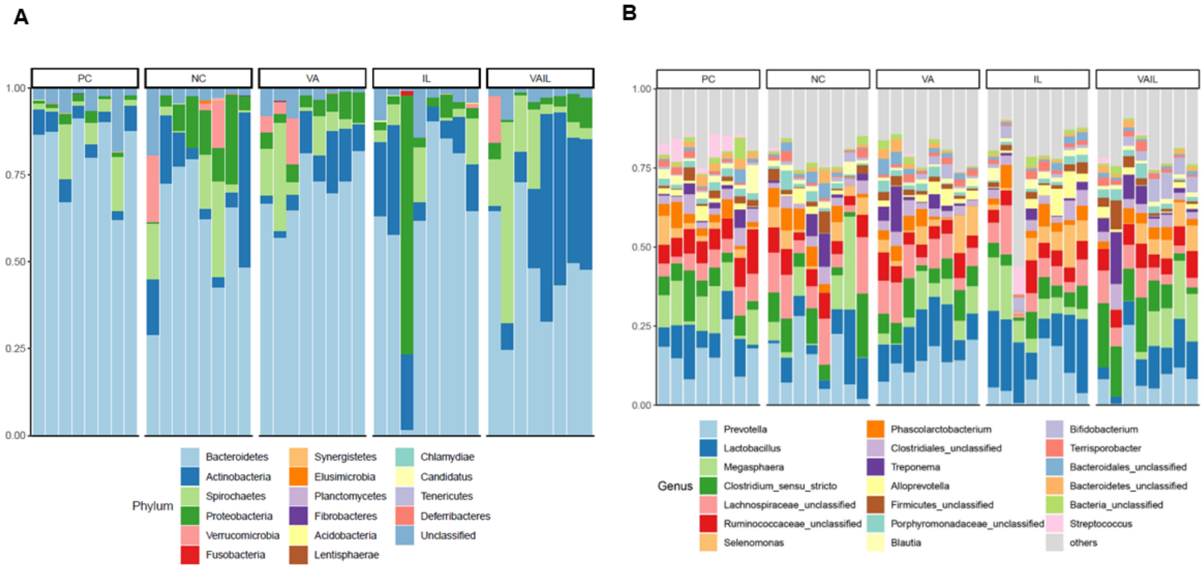

Figure S3. The relative abundance of colon bacterial composition at (**A**) phylum, and (**B**) genus levels for individual pigs fed with very low protein diets supplemented with isoleucine (Ile) at NRC and valine (Val) above NRC levels, or a combination of the two. PC: positive control, standard protein diet; NC: negative control, very low protein diet containing first four limiting amino acids (*i.e*., lysine, methionine, threonine, and tryptophan) at NRC (2012) levels; VA: NC containing Val above NRC level; IL: NC containing Ile at NRC level; VAIL: NC containing Val above NRC and Ile at NRC levels. *n* = 8 for each dietary group.

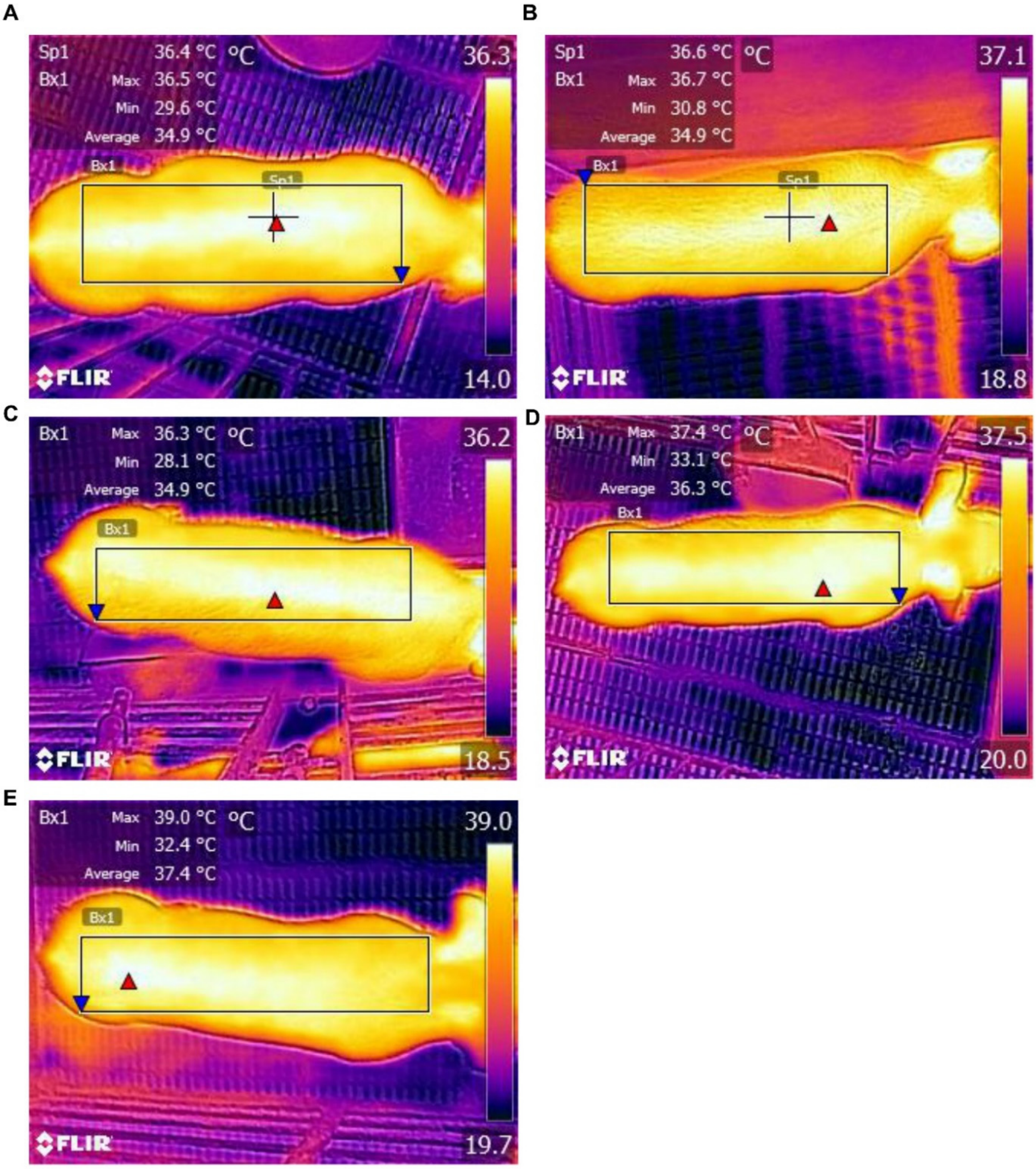

Figure S4. Representative thermal images of nursery pigs fed with very low protein diets supplemented with isoleucine (Ile) at NRC and valine (Val) above NRC levels, or a combination of the two. FLIR camera software (FLIR Research Studio, Wilsonville, OR) was used to calculate the mean of dorsal body surface temperature by drawing a rectangle from shoulder to rump. (**A**) PC, (**B**) NC, (**C**) VA, (**D**) IL, and (**E**) VAIL. PC: positive control, standard protein diet; NC: negative control, very low protein diet containing first four limiting amino acids (i.e., lysine, methionine, threonine, and tryptophan) at NRC (2012) levels; VA: NC containing Val above NRC level; IL: NC containing Ile at NRC level; VAIL: NC containing Val above NRC and Ile at NRC levels.
